# Effect of coffee and cocoa-based confectionery containing coffee on markers of cardiometabolic health: results from the pocket-4-life project

**DOI:** 10.1007/s00394-020-02347-5

**Published:** 2020-07-29

**Authors:** Daniela Martini, Alice Rosi, Michele Tassotti, Monica Antonini, Margherita Dall’Asta, Letizia Bresciani, Federica Fantuzzi, Valentina Spigoni, Raúl Domínguez-Perles, Donato Angelino, Cristian Ricci, Soledad Del Pozo-Luengo, Pedro Luis Tornel, Francesca Scazzina, Angel Gil-Izquierdo, Alessandra Dei Cas, Furio Brighenti, Riccardo Bonadonna, Daniele Del Rio, Pedro Mena

**Affiliations:** 1 Human Nutrition Unit, Department of Veterinary Sciences, University of Parma, University Hospital Building 27, Via Gramsci 14, 43126 Parma, Italy; 2 Department of Food, Environmental and Nutritional Sciences (DeFENS), Università degli Studi di Milano, 20122 Milan, Italy; 3 Human Nutrition Unit, Department of Food and Drugs, University of Parma, 43125 Parma, Italy; 4 Department of Medicine and Surgery, University of Parma, 43126 Parma, Italy; 5 Department of Animal Science, Food and Nutrition, Università Cattolica del Sacro Cuore, 29122 Piacenza, Italy; 6 Research Group on Quality, Safety and Bioactivity of Plant Foods, Department of Food Science and Technology, CEBAS-CSIC, University Campus of Espinardo, Edif. 25, 30100 Murcia, Spain; 7 Faculty of Bioscience and Technology for Food, Agriculture and Environment, University of Teramo, 64100 Teramo, Italy; 8 Pediatric Epidemiology, Department of Pediatrics, Medical Faculty, Leipzig University, Leipzig, Germany; 9 Clinical Analysis Service, University Hospital Virgen de la Arrixaca, 30120, El Palmar, Murcia, Spain; 10 School of Advanced Studies on Food and Nutrition, University of Parma, 43121 Parma, Italy

**Keywords:** Coffee, Cardiometabolic health, Dietary pattern, Randomized-controlled trial, Real-life setting

## Abstract

**Purpose:**

Coffee is an important source of bioactive compounds, including caffeine, trigonelline, and phenolic compounds. Several studies have highlighted the preventive effects of coffee consumption on major cardiometabolic (CM) diseases, but the impact of different coffee dosages on markers of CM risk in a real-life setting has not been fully understood. This study aimed to investigate the effect of coffee and cocoa-based confectionery containing coffee consumption on several CM risk factors in healthy subjects.

**Methods:**

In a three-arm, crossover, randomized trial, 21 volunteers were assigned to consume in a random order for 1 month: 1 cup of espresso coffee/day, 3 cups of espresso coffee/day, and 1 cup of espresso coffee plus 2 cocoa-based products containing coffee, twice per day. At the last day of each treatment, blood samples were collected and used for the analysis of inflammatory markers, trimethylamine *N*-oxide, nitric oxide, blood lipids, and markers of glucose/insulin metabolism. Moreover, anthropometric parameters and blood pressure were measured. Finally, food consumption during the interventions was monitored.

**Results:**

After 1 month, energy intake did not change among treatments, while significant differences were observed in the intake of saturated fatty acids, sugars, and total carbohydrates. No significant effect on CM markers was observed following neither the consumption of different coffee dosages nor after cocoa-based products containing coffee.

**Conclusions:**

The daily consumption of common dosages of coffee and its substitution with cocoa-based products containing coffee showed no effect on CM risk factors in healthy subjects.

**Trial registration number:**

Registered at clinicaltrials.gov as NCT03166540, May 21, 2017.

**Electronic supplementary material:**

The online version of this article (10.1007/s00394-020-02347-5) contains supplementary material, which is available to authorized users.

## Introduction

Coffee is one of the most consumed beverages, with about 1.5 billion cups daily consumed worldwide [1]. Despite the fact that, among the hundred described species pertaining to the genus *Coffea*, only two coffee species (i.e., *Coffea arabica* and *Coffea canephora*) are used to prepare coffee, there are many different ways to prepare coffee beverages, such as percolation, filtration, infusion, and others. From a chemical point of view, coffee is a complex mixture of several bioactive compounds. These include alkaloids, among which caffeine and trigonelline, phenolic compounds, mostly chlorogenic acids (CGAs), diterpenes (i.e., cafestol and kahweol), and melanoidins. The amount of all these bioactives in coffee widely vary based on coffee species as well as on coffee roasting, extraction, and brewing procedures [2,3].

In observational studies, coffee consumption has been inversely associated with risk of cardiovascular diseases, type-2 diabetes, and metabolic syndrome, in a dose–response manner [4,5]. A recent umbrella review considering 128 meta-analyses of observational and randomized-controlled trials (RCTs) showed that coffee, most probably due to caffeine and other bioactive compounds, might play a role in the prevention of several chronic diseases [6]. The potential mechanisms underneath the biological effects of coffee are multiple and not yet fully described. Among the bioactives, CGAs have been hypothesized to exert a specific competitive inhibition of the glucose-6-phosphate translocase in rat liver microsomes [7] or, at cellular level, they are able to activate adenosine monophosphate-activated protein kinase [8], leading to a regulation of the blood glucose homeostasis and inhibition of lipid synthesis. Furthermore, it has been shown that CGAs and trigonelline can negatively interfere with the gene expression of pathways involving NADPH oxidases and electron chain transport in the mitochondria, leading to an improvement of insulin sensitivity [9]. CGAs’ supplementation in diabetic rats was associated with a significant decrease of plasma of lipids, LDL- and total cholesterol, and lipid-related enzymes, possibly by a direct action on enzymes such as 3-hydroxy 3-methylglutaryl coenzyme A and lecithin cholesterol acyl transferase [10].

Despite evidence coming from animal and epidemiological research, not many RCTs have confirmed the role of coffee consumption on markers of the major cardiometabolic (CM) diseases. A meta-analysis of 15 RCTs of at least 1-week duration did not show any statistically significant effect of coffee consumption on blood pressure (BP) or the risk of hypertension [11]. These data on BP have been confirmed by a more recent umbrella review of meta-analyses focusing on intervention studies, while significant increases were found in total cholesterol, low-density lipoprotein (LDL) cholesterol, and triglycerides [6]. These results may be biased by the improper measurements of specific outcome (i.e., the self-evaluation of blood pressure), by the small number of outcomes, and by important confounders, i.e., smoking habits, that were not considered in the analysis. Moreover, in RCTs, coffee has often been provided for short periods, with different coffee doses and different coffee types,moreover, detailed information in the content of bioactive compounds is usually lacking, hindering the identification of potential compounds responsible for the observed effects. In addition, despite coffee shows dose-dependent responses in its associations with the risk of developing CM diseases [4,5,12], there are no RCTs assessing the effect of realistic patterns of low coffee consumption on key markers of CM risk.

Cocoa and chocolate are other foods that have been widely studied for the potential role on CM health, although only a weak evidence suggests that chocolate consumption may be associated with favorable health outcomes, including a reduced risk of cardiovascular diseases and diabetes [13]. Among potential mechanisms, cocoa and chocolate have been hypothesized to improve flow-mediated dilation, insulin, and HOMA-IR, despite there is a need for long-term studies [14] to fully confirm this.

Based on these premises, the aim of the present work was to investigate the effect of chronic consumption of different levels of coffee or cocoa-based confectionary containing coffee on a wide panel of CM risk factors in healthy subjects. In addition, since, for some individuals, the consumption of more than 1–2 coffees daily as beverage is unlikely, the substitution of coffee with cocoa-based products containing coffee was also considered. This may be an alternative strategy to supply coffee bioactives and to ensure their putative bioactivity. This work is part of a bigger study having as primary outcome the daily average concentration of a coffee-derived plasma circulating phenolic metabolites following the consumption of different doses of coffee and after its partial replacement with cocoa-based confectionary containing coffee.

## Materials and methods

### Products

To standardize brewing method and cup volume (approximately 35 mL), volunteers were provided with a single-serve coffee machine (Essenza EN 97.W, De'Longhi Appliances S.r.l, Treviso, Italy) and coffee capsules (Capriccio, Nespresso Italia S.p.a., Assago, Italy). Volunteers were also supplied with a cocoa-based product containing coffee commercially available (Pocket Coffee, Ferrero Commerciale Italia S.r.l., Alba, Italy).

The coffee capsules were selected based on their bioactive content after profiling several espresso coffee capsules available in the Italian market [15]. Particularly, coffee capsules used during the study provided an average of 74 mg/serving of caffeine, 11 mg/serving of theobromine, 75 mg/serving of trigonelline, 4 mg/serving of *N*-methylpyridinium, and 73 mg/serving of CGAs (59% corresponding to caffeoylquinic acids, while 41% to other hydroxycinnamates) (values as means of 9 capsules consumed throughout the intervention). The number of cocoa-based confectionery containing coffee was chosen, considering that the amount of caffeine provided by a cup of espresso coffee doubles approximately the amount of caffeine provided by two cocoa-based products containing coffee. Actually, a serving of 2 cocoa-based products containing coffee provided 27 mg of caffeine, 46 mg of theobromine, 0.1 mg of trigonelline, 0.5 mg/serving of *N*-methylpyridinium, and 8 mg of CGAs (77% corresponding to caffeoylquinic acids, while 23% to other hydroxycinnamates). Energy, nutrient, and bioactive content of coffee and cocoa-based products containing coffee are reported in Supplementary Table 1.

### Participants

Twenty-one healthy volunteers were recruited in the city of Parma, Italy, through announcements placed at the university, the hospital, and other public places. All subjects potentially involved in the intervention were informed of the details of the protocol. Those who agreed to participate were asked to sign a written informed consent before enrollment in the study. Information to the volunteers was provided before and separately from the consent form.

Inclusion criteria were being aged > 18 years, healthy, of normal weight [body mass index (BMI) 18–25 kg/m^2^], and regular consumers of one to five cups of coffee per day. Exclusion criteria were clinical diagnosis for metabolic, renal or digestive disorders, regular consumption of medication for chronic diseases, current use of supplements (e.g., vitamins, minerals), antibiotic therapy taken within the last 3 months, intense physical activity (highly active subjects who declared to do vigorous physical activity > 5 times a week and > 2 h/time), pregnancy, or lactation. To avoid likely confounding factors, subjects who declared regular intake of coffee exceeding five coffees per day, and very high consumption of coffee/cocoa-related phytochemicals were not considered suitable and were excluded from the study. No restrictions were instead used for smoking status.

### Study design and protocol

The study protocol was approved by the Ethics Committee for Human Research for Parma Hospital and University (AZOSPR/0015693/6.2.2.) and was registered at clinicaltrials.gov on May 21, 2017 (as NCT03166540). Volunteers were recruited and enrolled between December 2016 and January 2017, and the study was completed in May 2017.

The study design has been previously published [16]. Briefly, in a three-arm, crossover, randomized trial, 21 volunteers were assigned to consume three treatments in a random order for 1 month: 1 cup of espresso coffee/day (hereinafter named “1C group”), 3 cups of espresso coffee/day (“3C group”), and 1 cup of espresso coffee plus 2 cocoa-based products containing coffee twice per day (“PC group”). Randomization list was generated using Random Number Generator Pro (Segobit Software).

Minimal recommendations to avoid other dietary sources of coffee/cocoa-related phytochemicals besides what introduced through the assigned treatment were provided for the 2 days prior to and during the sampling day, by providing subjects a list of allowed and prohibited foods to be consumed. Moreover, to standardize the time of coffee consumption, subjects were instructed to consume coffee(s) at specific time for the 2 days prior to each sampling day and on the sampling day. In addition, participants were asked to maintain their habitual lifestyle during the whole intervention period, to not change their habitual physical activity and diet (except for the study constraints). The day before the sampling day, time of dinner and its composition were standardized (i.e., subjects were asked to consume the same dinner the day before each sampling day, choosing from foods present in the above-mentioned list of permitted foods) and only water was allowed as a drink overnight. Subjects were asked to have dinner no later than 8:00 PM the day before the sampling day. The following morning of the sampling day, subjects attended the ambulatory of the Endocrinology Unit of the Department of Medicine and Surgery at the University of Parma, where fasting blood sampling (t0) and medical visit were carried out. Baseline blood samples were collected, and anthropometric characteristics and blood pressure were measured, as detailed below. After that, all subjects consumed (at about 9.00 A.M.) a cup of coffee, together with a low-phenolic breakfast (a sponge milky cake). This was the only dose of coffee for treatment 1C, whereas treatment 3C consumed two more cups of coffee (at 12.00 noon and 3.00 P.M.) (without sugar, sweeteners, and milk for the first coffee), while treatment PC consumed two cocoa-based products containing coffee twice during the day (at 12.00 noon and 3.00 P.M). Starting just after the consumption of the morning coffee, blood samples were collected at selected time points (t 0.5, t1, t2, t3, t4, t5, t6, t7, t8, and t9), as previously reported [16]. Five hours after the consumption of the morning coffee, participants received a standardized lunch (ham and cheese sandwich plus mineral water) free of coffee/cocoa-related phytochemicals. Water was available ad libitum during the whole sampling day. Subjects left the clinic just at 6.00 P.M. after the last sampling of the first day (t 9), and were asked to return the day after for the last sampling (t 24). Twenty-four hours after receiving the first coffee of the sampling day, blood samples were indeed taken to check for return to baseline of the circulating phytochemicals. Samples collected at 24 h were used for the analysis of biomarkers included in the present manuscript. After the 24-h sampling, subjects could leave the clinic and continue to consume 1C, 3C, or PC for 1 month, based on the randomization protocol.

### Nutritional assessment

The dietary intake of the volunteers and the compliance to the study during the intervention periods were assessed using a 3-day weighed food diary (2 weekdays and a weekend day) for each intervention arm. Moreover, compliance was also assessed through a second round of food diaries filled in the 2 days before and during the sampling day, that were also used to verify that subjects consumed a standardized dinner. After the enrollment, a nutritionist trained each participant in the use of the food diary, to obtain a complete description of all foods and beverages consumed during the day. Participants were also requested to record the weight of each food/beverage consumed by weighing the product or, if not possible, by evaluating the portion size through standard household measures and a food atlas [17]. At the end of each recording period, a nutritionist checked the accuracy of the information registered in the food diary in the presence of the participant.

Daily intake of foods was directly calculated through the food diaries, while daily intakes of energy and macronutrients were estimated by linking food and beverage consumption with the food database of the European Institute of Oncology [18] through an in-house Microsoft Access® application.

Compliance was calculated by dividing the number of coffee capsule and/or cocoa-based products containing coffee consumed (as declared in the food diaries) by the number of days which the products should have been consumed and then multiplying by 100.

### Anthropometric measurement

Height was measured during the enrollment phase with a stadiometer (Seca 200, Seca, Hamburg, Germany). Weight was measured at every visit, with subjects wearing light clothing and without shoes to the nearest 0.1 kg on a mechanical column scale (Seca 700, Seca, Hamburg, Germany). Height and weight were used to measure the BMI, expressed as kg/m^2^. Waist circumference was measured during every visit at the midpoint between the lower margin of the least palpable rib and the top of the iliac crest, while the subject was standing, using a stretch-resistant tape. To avoid subjective errors, all these measurements were performed by the same operator.

### Blood pressure monitoring

Systolic (SBP) and diastolic blood pressure (DBP) were measured in the morning of the beginning and end of each intervention period.

After a 10-min rest in a seated position in a quiet room, BP was measured at the right upper arm by a physician, using a digital blood pressure monitor with 1 mmHg precision (Citizen Systems Japan Co., LTD).

### Biochemical analyses

Blood was obtained from an intravenous catheter into tubes containing EDTA, heparin, or nothing depending on the analysis. Blood samples were centrifuged at 2400*g* × 10 min at 4 °C, and supernatants were aliquoted and stored at − 80 °C until further processing.

Serum total, HDL- and LDL-cholesterol, as well as triglycerides, were determined by standardized routine hospital protocols.

Glycaemia was determined in serum by an automatic clinical analyser with a combined enzymatic-electrochemical system (YSI 2900 STAT PLUS, Yellow Springs Instruments, Yellow Springs, Ohio). Serum insulin concentration was monitored using routine blood analysis, while fasting insulin sensitivity was determined using the quantitative insulin sensitivity check index (QUICKI) [19]. Fasting insulin secretion capacity was evaluated as the Homeostatic Model Assessment for Insulin Resistance (HOMA-IR) according to the related equation [20].

Plasma nitric oxide, assessed as nitrate/nitrite, was determined using Nitrate/Nitrite Fluorometric Assay Kit (Cayman Chemical, Ann Arbor, MI, USA).

Trimethylamine-*N*-oxide (TMAO) was determined in plasma by means of a UHPLC-MS/MS method. Samples were defrosted at room temperature, shaken, diluted 1:10 with acetonitrile and centrifuged at 13,765*g* for 10 min before being transferred in vials for the analysis. Analysis of extracted plasma was performed using an Accela UHPLC 1250 equipped with linear ion trap-mass spectrometer (LTQ XL, Thermo Fisher Scientific Inc., San Jose, CA, USA) fitted with a heated-electrospray ionization (H-ESI-II) probe (Thermo Fisher Scientific Inc., San Jose, CA, USA). Separation was carried out by means of an Xbridge BEH HILIC XP (100 × 2.1 mm) with a porosity of 2.5 μm (Waters, USA). Quantification was carried out by comparison with the reference standard [21].

Finally, interleukin 8 (IL-8), tumor necrosis factor (TNFα), and vascular endothelial growth factor (VEGF), as specific markers associated with inflammatory processes, were analyzed in plasma with Human High Sensitivity Cytokine 3-Plex customized Immunoassay (R&D System, Inc. CA) using Luminex Technology (Bio-Plex® MAGPIX™ Multiplex Reader; Bio-Rad Laboratories S.r.l., Italy). Determination of biomarkers was performed according to the instruction of the manufacturer.

### Statistical analysis

As previously reported [16], the sample size was calculated by considering as primary outcome the daily average concentration of a coffee-derived plasma circulating phenolic metabolite. In detail, 15 subjects had to complete an acute intervention to detect a change of 600 nmol/h/L^−1^ in dihydrocaffeic acid-3′-sulfate plasma concentration with a standard deviation (SD) of 870 nmol/h/L^−1^ (α error of 0.05, 80% power, and two-sided testing). Sample size was not calculated for secondary outcomes. However, the sample size was preliminarily estimated as appropriate for all the secondary outcomes, since it reflected the sample size considered in other RCTs that evaluated the effects of coffee intake on the same markers [22–24]. A post hoc calculation was also carried out to understand the actual power for each variable.

The Kolmogorov–Smirnov test was used to evaluate the normality of data distribution. Food intakes are presented as median and interquartile range. Data were then analyzed using the Friedman non-parametric test to explore differences among the three treatment groups, and Wilcoxon non-parametric tests for between-group comparisons. Nutritional variables (energy and nutrients) are reported as mean ± SD while CM markers as mean ± SEM. Repeated measurement General Linear Model (GLM) was performed to assess the effect of treatment on energy, nutrients, and CM markers, testing the assumption of sphericity through the Mauchly’s test and using Greenhouse–Geisser corrections if epsilon was lower than 0.75 or Huynh–Feldt corrections if epsilon was greater than 0.75, when the assumption of sphericity was violated. In addition, if a main effect of treatment was registered. The same model was used to test the effect of treatment on CM markers accounting for sex and smoking habits as confounding factors. Carry-over effects were investigated by means of two separate analyses. First, the effects given by two opposite treatment sequence were compared [25] (ref). Afterwards, the effects given by treatment sequence were estimated and a Wald test on the test coefficient of the interaction term between treatment and time was performed. Bonferroni post hoc tests were used for multiple comparisons in all of the above evaluations. A difference was considered significant at *p* < 0.05. The statistical analysis was performed with the Statistical Package for Social Sciences software (IBM SPSS^®^ Statistics, Version 23.0. IBM Corp., Chicago, IL).

## Results

### Characteristics of participants

All the 21 participants (10 males and 11 females) completed the study. Eight out of the 21 subjects were smokers, while 13 were non-smokers. The main characteristics of the subjects at baseline are reported in Table 1. Subjects had BMI and all the CM markers within normal ranges.

**Table 1:** Demographic and clinical characteristics of study participants at baseline (*n* = 21; mean ± SEM)

	All participants (*n* = 21)
Age (years)	22.9 ± 0.5
Sex (female/males)	11/10
Habitual coffee consumption (serving/day)	2.3 ± 0.2
Smoking habits (smokers/non-smokers)	8/13
Physical activity (MET-minute/day)	1809 ± 387
Body weight (kg)	67.0 ± 2.7
Body mass index (kg/m^2^)	22.3 ± 1.7
Waist circumference (cm)	72.0 ± 2.7
Systolic blood pressure (mmHg)	116.6 ± 1.7
Diastolic blood pressure (mmHg)	73.7 ± 6.6
Total cholesterol (mg/dL)	173.4 ± 5.4
LDL-cholesterol (mg/dL)	91.4 ± 3.7
HDL-cholesterol (mg/dL)	65.7 ± 4.5
Triglycerides (mg/dL)	81.8 ± 1.2
Fasting blood glucose (mg/dL)	85.3 ± 4.0
Fasting insulin (μU/mL)	11.0 ± 0.9
HOMA-IR	2.4 ± 0.9
QUICKI	0.36 ± 1.5
Nitric oxide (μmol/L)	13.0 ± 0.3
TMAO (μmol/L)	3.1 ± 1.2
IL-8 (pg/mL)	9.2 ± 0.7
VEGF (pg/mL)	60.2 ± 12.8

*HDL* high-density lipoprotein, *HOMA-IR* homeostatic model assessment for insulin resistance, *IL-8* interleukin-8, *LDL* low-density lipoprotein, *QUICKI* quantitative insulin sensitivity check index, *TMAO* trimethylamine *N*-oxide, *TNF-α* tumor necrosis factor *α*, *VEGF* vascular endothelial growth factor

### Compliance and nutritional assessment

No adverse events were reported among volunteers during the whole-study period. The analysis of food diaries showed that participants approximately maintained the same diet over the study period, regardless of the assigned treatment. There was no main effect of treatment on the daily intake of the majority of the food groups, while, as expected, differences were observed for the “coffee” and the “dessert & confectionary” groups (Table 2). Indeed, volunteers consumed three times more coffee in the 3C period (105 mL/day), corresponding to three espresso coffees a day, than in the 1C and PC months (35 mL/day), during which they had only one espresso coffee a day. Moreover, significant differences were registered among the three treatment groups for the intake of “dessert & confectionary”, because, during the PC treatment, volunteers had around 50 g per day of cocoa-based products containing coffee that were apparently not eaten as a replacement of habitual dessert intake, but instead in addition to it. Thus, dessert and confectionary intake was higher during the PC treatment than in the other two treatments (Table 2).

**Table 2:** Food groups by treatment group (*n* = 21)

Parameters	1C		3C		PC		*p* value
Coffee (mL/day)	35.00	(0.00)^b^	105.00	(0.00)^a^	35.00	(0.00)^b^	0.000
Tea, herbal tea, and infusion (mL/day)	0.00	(49.17)	3.33	(70.00)	0.00	(58.33)	0.061
Alcoholic beverage (mL/day)	100.00	(250.00)	193.33	(336.67)	133.33	(227.33)	0.784
Non-alcoholic beverage (mL/day)	0.00	(0.00)	0.00	(66.67)	0.00	(0.00)	0.307
Juice (mL/day)	0.00	(30.00)	0.00	(66.67)	0.00	(36.67)	0.982
Fruit (g/day)	109.67	(156.83)	99.67	(139.17)	120.00	(130.83)	0.784
Nuts (g/day)	0.00	(8.17)	0.05	(15.87)	2.50	(6.67)	0.724
Spices and herbs (g/day)	0.00	(2.08)	0.00	(0.30)	0.00	(1.83)	0.359
Vegetable (g/day)	135.33	(105.33)	86.67	(117.11)	93.33	(101.67)	0.276
Tuber and potato (g/day)	0.00	(21.67)	17.00	(30.00)	16.67	(45.33)	0.220
Bread, crispbread, and rusk (g/day)	105.00	(86.83)	148.33	(114.42)	151.33	(119.85)	0.717
Pasta and other cereals (g/day)	50.15	(42.49)	77.54	(75.55)	74.97	(58.20)	0.110
Breakfast cereal (g/day)	0.00	(4.17)	0.00	(6.67)	0.00	(9.67)	0.798
Legumes (g/day)	0.00	(28.33)	11.67	(23.33)	0.00	(25.00)	0.258
Milk and yogurt (g/day)	83.33	(100.07)	66.67	(87.62)	71.60	(68.00)	0.592
Dairy (g/day)	38.33	(40.03)	36.67	(40.48)	33.33	(36.73)	0.229
Egg (g/day)	0.00	(35.83)	1.20	(20.83)	0.00	(19.50)	0.743
Meat (g/day)	43.33	(54.67)	26.67	(56.67)	33.33	(64.35)	0.627
Cold cut and preserved meat (g/day)	23.33	(25.83)	23.33	(31.50)	26.67	(21.67)	0.640
Fish and shellfish (g/day)	16.67	(39.00)	13.33	(48.33)	16.67	(52.50)	0.625
Oil and fat (g/day)	19.30	(15.20)	17.94	(17.37)	20.00	(20.82)	0.864
Dessert and confectionary (g/day)	84.33	(69.73)^b^	48.04	(64.05)^c^	129.77	(80.55)^a^	0.000
Miscellaneous (g/day)	3.67	(12.84)	0.67	(7.74)	1.15	(7.60)	0.368

All values are reported as median (IR). Different letters in the same row indicate differences among the three treatment groups (*p* < 0.05 from repeated measurement Friedman non-parametric tests with pairwise post hoc test)

Legend: 1C: group consuming 1 cup of espresso coffee/day; 3C: group consuming 3 cups of espresso coffee/day; PC: group consuming 1 cup of espresso coffee plus 2 cocoa-based products containing coffee twice per day.

On the basis of reported consumption of test products with the use of food diaries, the median compliance was 100% in the three intervention arms.

In agreement with the consumption of food groups, no clear main effect (*p* = 0.067) of the treatment on average energy intake was observed (Table 3). Actually, the daily energy intake did not significantly change among the three treatment periods, although a higher intake was registered during the PC period. This increased intake of around 200 kcal per day for this treatment may be attributed to the consumption of the cocoa-based confectioneries containing coffee (220 kcal/day). Regarding macronutrients, the daily intake of total proteins and total fats were not affected by the treatment, while a higher intake of total carbohydrates was observed during the PC period. This is likely due to an increased intake of sugars present in the cocoa-based confectioneries containing coffee (28.3 g/day) and not in the coffee. Similarly, the saturated fatty acid intake was higher during the PC treatment compared to 1C and 3C (Table 3), being 6.6 g the daily amount of saturated fatty acids provided by the confectionary product. Accordingly, energy from sugars and saturated fatty acids was higher during the PC period compared to 1C and 3C treatments (Table 3).

**Table 3:** Energy and nutrient intakes by treatment group (*n* = 21)

Parameters	1C	3C	PC	*p* value
Energy (kcal/day)	1860 ± 551	1997 ± 610	2127 ± 529	0.067
Total protein (g/day)	68.8 ± 25.9	65.6 ± 16.2	62.8 ± 16.2	0.278
Animal protein (g/day)	44.0 ± 19.5	37.3 ± 13.2	38.1 ± 15.4	0.122
Vegetable protein (g/day)	24.5 ± 10.9	28.1 ± 8.9	24.6 ± 6.8	0.095
Total fat (g/day)	74.1 ± 26.8	75.3 ± 29.3	85.6 ± 24.7	0.061
Animal fat (g/day)	40.6 ± 16.4	38.7 ± 15.1	44.4 ± 16.4	0.127
Vegetable fat (g/day)	33.5 ± 16.9	36.5 ± 21.2	41.1 ± 15.1	0.197
Cholesterol (mg/day)	244 ± 114	216 ± 92	218 ± 79	0.497
Saturated fatty acids (g/day)	24.0 ± 9.6^b^	25.1 ± 9.3^b^	31.6 ± 8.5^a^	0.000
Available carbohydrates (g/day)	210.3 ± 71.9^b^	237.6 ± 66.7^ab^	250.7 ± 65.8^a^	0.028
Soluble carbohydrates (g/day)	75.1 ± 29.8^b^	75.1 ± 26.4^b^	103.1 ± 25.9^a^	0.000
Dietary total fiber (g/day)	18.0 ± 11.4	20.3 ± 8.5	17.3 ± 6.5	0.253
*% Total energy from*				
Total protein	14.7 ± 2.9^a^	13.6 ± 2.5^ab^	12.1 ± 2.6^b^	0.002
Animal protein	9.4 ± 3.3^a^	7.9 ± 2.9^ab^	7.4 ± 2.9^b^	0.030
Vegetable protein	5.3 ± 1.3^ab^	5.7 ± 0.8^a^	4.7 ± 0.9^b^	0.001
Total fat	35.6 ± 7.3	33.6 ± 5.8	36.3 ± 6.6	0.215
Animal fat	19.7 ± 6.5	17.7 ± 5.5	18.8 ± 5.8	0.333
Vegetable fat	15.9 ± 6.0	15.8 ± 5.6	17.5 ± 4.7	0.468
Saturated fatty acids	12.1 ± 3.5^ab^	11.3 ± 2.5^b^	13.5 ± 2.4^a^	0.010
Available carbohydrates	45.4 ± 6.5	48.1 ± 6.3	47.4 ± 5.9	0.226
Soluble carbohydrates	16.4 ± 4.9^b^	15.2 ± 3.7^b^	19.6 ± 2.9^a^	0.000
Dietary total fiber	1.9 ± 0.7^ab^	2.0 ± 0.6^a^	1.6 ± 0.4^b^	0.022

All values are reported as mean ± SD. Different letters in the same row indicate differences among the three treatment groups (p < 0.05 from repeated measurement GLM with Bonferroni post hoc test)

Legend: 1C: group consuming 1 cup of espresso coffee/day; 3C: group consuming 3 cups of espresso coffee/day; PC: group consuming 1 cup of espresso coffee plus 2 cocoa-based products containing coffee twice per day

### Markers of cardiometabolic health

Table 4 shows the values of the anthropometric and CM markers of the 21 subjects after the three interventions (1C, 3C, and PC). There were no significant changes in the anthropometric measures (i.e., body weight, BMI, and waist circumference) and BP (both SBP and DBP) values following any of the intervention arms. Similarly, the interventions did not impact parameters linked to blood glucose metabolism (i.e., fasting glycaemia and insulinemia, HOMA-IR, and QUICKI) or to lipid metabolism (HDL-, LDL- and total cholesterol, and triglycerides). Furthermore, inflammatory markers, such as nitric oxide, IL-8, TNF-α, VEGF, as well as TMAO, were not significantly different among the three intervention groups. Similarly, no effect of treatment on CM markers was observed when sex and smoking habits were considered as confounding factors (data not shown). Supplementary Table 2 reports about the carry-over effects. Comparisons between effects among different treatment sequences were not statistically significant. Finally, no statistically significant effects associated with the treatment sequence were observed (Supplementary Table 3).

**Table 4:** Cardiometabolic markers by treatment group (*n* = 21)

Parameters	1C	3C	PC	*p *value
Body weight (kg)	67.1 ± 2.8	67.1 ± 2.7	67.1 ± 2.7	0.998
BMI (kg/m^2^)	22.4 ± 0.6	22.4 ± 0.5	22.4 ± 0.5	0.995
Waist circumference (cm)	73.2 ± 1.7	73.4 ± 1.8	73.7 ± 1.7	0.568
Systolic BP (mmHg)	113.7 ± 2.6	115.5 ± 2.1	113.0 ± 2.2	0.584
Diastolic BP (mmHg)	73.1 ± 1.6	72.3 ± 1.2	71.1 ± 1.9	0.589
Total cholesterol (mg/dL)	166.0 ± 6.6	170.1 ± 6.3	168.0 ± 5.9	0.534
LDL-cholesterol (mg/dL)	87.9 ± 5.6	87.5 ± 5.6	86.0 ± 5.1	0.788
HDL-cholesterol (mg/dL)	65.3 ± 3.1	65.3 ± 3.6	65.9 ± 3.7	0.948
Triglycerides (mg/dL)	83.1 ± 6.0	85.1 ± 5.2	80.7 ± 5.5	0.657
Fasting blood glucose (mg/dL)	87.4 ± 1.2	86.8 ± 1.0	85.6 ± 1.2	0.372
Fasting insulin (μU/mL)	10.8 ± 1.2	8.8 ± 0.8	8.9 ± 0.6	0.174
HOMA-IR	2.4 ± 0.3	1.9 ± 0.2	1.9 ± 0.1	0.157
QUICKI	0.34 ± 0.01	0.36 ± 0.01	0.35 ± 0.00	0.378
NO (μmol/L)	11.7 ± 1.7	11.3 ± 1.7	12.7 ± 1.6	0.757
TMAO (μmol/L)	2.5 ± 0.3	2.8 ± 0.3	2.5 ± 0.2	0.573
IL-8 (pg/mL)	9.3 ± 1.1	9.9 ± 1.2	10.4 ± 1.3	0.357
TNFα (pg/mL)	8.0 ± 0.7	8.0 ± 0.8	8.1 ± 0.8	0.921
VEGF (pg/mL)	59.1 ± 11.8	69.2 ± 15.6	60.1 ± 13.5	0.415

*BMI* body mass index, *BP* blood pressure, *HDL* high-density lipoprotein, *HOMA-IR* homeostatic model assessment for insulin resistance, *IL-8* interleukin-8, *LDL* low-density lipoprotein, *NO* nitric oxide, *QUICKI* quantitative insulin sensitivity check index, *TMAO* trimethylamine *N*-oxide, *TNF-α:* tumor necrosis factor*-α*, *VEGF* vascular endothelial growth factor, Legend: 1C: group consuming 1 cup of espresso coffee/day; 3C: group consuming 3 cups of espresso coffee/day; PC: group consuming 1 cup of espresso coffee plus 2 cocoa-based products containing coffee twice per day

All values are reported as mean ± SEM

## Discussion

The present work was carried out to examine the CM effects of the daily consumption of coffee and cocoa-based confectionery containing coffee in healthy subjects. Results suggested that no effects, neither beneficial nor detrimental, were measurable on the considered CM outcomes after 1-month consumption of two different common coffee dosages (1 coffee and 3 coffees daily) or after the partial substitution of 2 coffee servings with cocoa-based products containing coffee. Food consumption and energy intake did not change throughout the intervention. As expected, the only exceptions were for coffee and for the category “sweet and confectionary intake” which include the test foods used in the intervention (i.e., coffee and cocoa-based products containing coffee).

The effect of coffee consumption on CM markers has been extensively investigated in both observational and intervention studies. Overall, studies seem to suggest that coffee may be part of a healthy diet, providing in general more benefit than harm to human health, especially at doses of three–four cups a day [26]. About potential detrimental effects, coffee and mainly unfiltered coffee, has been found to potentially increase serum lipids, due to the significant content of diterpenes [6]. Conversely, both filtered and decaffeinated coffees showed negligible effects on blood lipids [26]. Despite this evidence, a thorough comparison with findings from the previous studies could be problematic, due to the high heterogeneity among studies in terms, for example, of variability of amount and types of coffees drunk or population characteristics. For instance, in a meta-analysis of 10 RCTs that did not find any significant effect of coffee on BP, it has been observed that six trials instructed participants to consume a minimum amount of coffee but no maximum, and only four used a standardized amount [11].

Concerning the variability of types of coffee, it has been demonstrated that the content of bioactive compounds, like caffeine and CGAs present in coffee, may drastically vary depending on factors like coffee species, degree of roasting, and coffee extraction procedure [2]. We tried to avoid this source of variability by providing to each subject capsules produced by the same company and containing exactly the same type of coffee, extensively characterized in a previous study [15]. We also verified that, despite three lots coffee products have been used during the whole-study duration, these lots were almost identical for type and quantity of compounds extracted (data not shown). However, this accurate chemical characterization is uncommon in RCTs, and therefore, it is hard to have a straightforward comparison among findings from different studies.

Besides the variation in terms of coffee dosage and bioactive contents, the characteristics of the subjects represent another major source of variations among different studies. For example, as shown in a recent review, the effect of hydroxycinnamic acids on CM risk factors may be greater in individuals at higher CM risk (e.g., with higher fasting glycaemia an cholesterol, BP) [27],however, the health status of subjects has not been considered in many RCTs. Among studies reporting the health status, two studies performed by the same group observed an improvement on some cardiovascular and metabolic syndrome markers in hypercholesterolemic subjects after 8-week consumption of a soluble/green roasted coffee [28,29]. Some benefits in CM markers were also observed for normocholesterolemic individuals and they might be related to the higher daily intake of CGAs (510 mg vs. 201 mg for the 3C group) and the longer duration of the study (8 weeks against 1 month) compared to the present study. Conversely, in another study performed on healthy subjects, the consumption of 3 or 5 cups/day of coffee for 8 weeks had no effects on several markers of oxidative stress, as well as on blood glucose, insulin, cholesterol, triglycerides, BP, and some inflammatory markers [12]. These results are in line with our study, despite daily intake of CGAs was much higher (365 and 607 mg/day for the three cup and the five cup groups, respectively).

Other than the health status, the inter-individual variability may affect human metabolism and bioavailability of bioactives, which, in turn, may influence the individual response to coffee compounds [30,31]. This variation may belong to (i) genetic factors, such as polymorphisms of *CYP1A2,* impacting caffeine metabolism by cytochrome P450 1A2 and leading to the clusterization of these subjects as slow or quick caffeine metabolizers; (ii) non-genetic factors, e.g., sex, age, smoking habits, pathophysiological status, and, above all, gut microbiota profile [31,32]. The main strategies to bypass these variabilities are the choice of an adequate sample size and a defined target population. These aspects might represent limitations of the present study, as 21 apparently healthy subjects might not have been the best target population to show any effect of coffee or coffee-containing product intake on CM risk factors. The sample size was calculated based on the daily mean concentration of a coffee-derived plasma circulating phenolic metabolite [16], but, nevertheless, it was similar to those of other RCTs evaluating the impact of coffee on markers of CM health [22–24]. Therefore, this sample size was consistent with the main scope of the present work, which was to evaluate the CM health effects of coffee/coffee-containing product consumption of a general healthy population in the frame of their daily habits. However, the post hoc power calculation performed to estimate the actual power for each variable revealed an underpower, which seems to be due not only to the small sample size but also to the very small effect sizes (Supplementary Table 4). Small effect sizes may be in line with the cardiometabolic status of the participants at the beginning of the intervention, young adults in good health. Other study limitation may be the short duration of the study (1-month per arm), which may have played a role in the lack of variations observed. However, this period appears to be consistent with the previous observations that some CM markers, like BP and blood lipid profile, tend to stabilize after about 4 weeks in response to fixed nutritional interventions, as assessed by the European Food Safety Authority [33].

The present study has also several strengths. First, the timing of coffee intake (morning, mid-morning, and after lunch) and the intake of coffee per day (from 1 to 3 cups) were selected to reproduce a realistic setting, since Italians consume, on average, 2–3 coffees per day [34]. Second, the study was carried out in free-living subjects, who kept their lifestyle unchanged along the trial, as confirmed by diaries related to physical activity and dietary habits. The will of performing the study in a real-life setting explains our decision to avoid run-in and wash-out periods [35]. Moreover, in addition to compliance to the intervention, dietary habits and lifestyle were carefully evaluated throughout the whole intervention period, by considering that most of the outcomes under study can be affected by many other dietary and behavioral habits.

## Conclusions

In conclusion, based on the present study, the consumption of one or three coffee per day or the substitution of two coffee servings per day with cocoa-based products containing coffee for 1 month had no effect of several CM risk factors in healthy subjects. Furthermore, well-powered and designed studies are warranted to better investigate the possible role of coffee consumption on CM risk or health maintenance in general. To properly address this aim, inter-individual variation in the study population and the true internal exposure of each subject to coffee bioactives should be fully considered.

## Electronic supplementary material

Below is the link to the electronic supplementary material.

Supplementary file1 (DOCX 31 kb)
