# Supplementary material for: Effect of coffee and cocoa-based confectionery containing coffee on markers of cardiometabolic health: results from the pocket-4-life project
Source: Eur J Nutr. 2020 Jul 29;60(3):1453–63. doi: 10.1007/s00394-020-02347-5 (PMC7987697; doi:10.1007/s00394-020-02347-5)
Supplement: Supplementary file 1 — Supplementary file1 (DOCX 31 kb) [file 394_2020_2347_MOESM1_ESM.docx]

**EFFECT OF COFFEE AND COCOA-BASED CONFECTIONERY CONTAINING COFFEE ON MARKERS OF CARDIOMETABOLIC HEALTH: RESULTS FROM THE POCKET-4-LIFE PROJECT**

Daniela Martini^1,2^, Alice Rosi^3^, Michele Tassotti^3^, Monica Antonini^4^, Margherita Dall’Asta^3,5^, Letizia Bresciani^1^, Federica Fantuzzi^4^, Valentina Spigoni^4^, Raúl Domínguez-Perles^6^, Donato Angelino^1,7^, Cristian Ricci^8^, Soledad Del Pozo-Luengo^9^, Pedro Luis Tornel^9^, Francesca Scazzina^3^, Angel Gil-Izquierdo^6^, Alessandra Dei Cas^4^, Furio Brighenti^3^, Riccardo Bonadonna^4^, Daniele Del Rio^1,10*^ and Pedro Mena^3^

^1^ Human Nutrition Unit, Department of Veterinary Sciences, University of Parma, 43126 Parma, Italy.

^2^ Department of Food, Environmental and Nutritional Sciences (DeFENS), Università degli Studi di Milano, 20122 Milan, Italy

^3^ Human Nutrition Unit, Department of Food & Drugs, University of Parma, 43125 Parma, Italy.

^4^ Department of Medicine and Surgery, University of Parma, 43126 Parma, Italy.

^5^ Department of Animal Science, Food and Nutrition, Università Cattolica del Sacro Cuore, 29122 Piacenza, Italy

^6^ Research Group on Quality, Safety and Bioactivity of Plant Foods. Department of Food Science and Technology, CEBAS-CSIC, University Campus of Espinardo, Edif. 25, 30100 Murcia, Spain

^7^ Faculty of Bioscience and Technology for Food, Agriculture and Environment, University of Teramo, 64100 Teramo, Italy

^8^ Pediatric Epidemiology, Department of Pediatrics, Medical Faculty, Leipzig University, Leipzig, Germany.

^9^ Clinical Analysis Service, University Hospital Virgen de la Arrixaca, 30120, El Palmar, Murcia, Spain.

^10^ School of Advanced Studies on Food and Nutrition, University of Parma, 43121 Parma, Italy

**Corresponding author:** Daniele Del Rio, University of Parma, University Hospital building 27, Via Gramsci 14, 43126 Parma, Italy. Email: [daniele.delrio@unipr.it](mailto:daniele.delrio@unipr.it)

Supplementary Table 1. Energy, nutrient and bioactive content of single coffee and cocoa-based product containing coffee, and of the three different treatments per day

| **Compounds** | **1 coffee (35 mL)** | **CBPCC (1 product =12.5 g)** | **Treatments** | | |
| --- | --- | --- | --- | --- | --- |
|  |  |  | **1C** | **3C** | **PC** |
| Energy (kcal) | ND | 55 | ND | ND | 220 |
| Protein (g) | ND | 0.4 | ND | ND | 1.6 |
| Carbohydrates (g) | ND | 7.4 | ND | ND | 29.6 |
| Sugar (g) | 5 | 7.1 | 5 | 15 | 43.4 |
| Fibre (g) | ND | 0.4 | ND | ND | 1.6 |
| Total fat (g) | ND | 2.6 | ND | ND | 10.4 |
| Saturated fatty acids (g) | ND | 1.6 | ND | ND | 6.4 |
| Sodium (mg) | ND | 2.3 | ND | ND | 9.2 |
| Flavan-3-ols^§^ (mg) | ND | 1.3 | ND | ND | 5.1 |
| Caffeoylquinic acids* (mg) | 43.2 | 6.3 | 43.2 | 129.6 | 68.5 |
| Other phenolic acids (mg) | 28.6 | 1.8 | 28.6 | 85.9 | 35.8 |
| Niacin (mg) | 0.47 | 0.06 | 0.47 | 1.41 | 0.70 |
| *N*-methylpyridinium (mg) | 4.17 | 0.48 | 4.17 | 12.51 | 6.11 |
| Trigonelline (mg) | 74.60 | 0.10 | 74.60 | 223.80 | 74.98 |
| Caffeine (mg) | 73.84 | 27.18 | 73.84 | 221.51 | 182.56 |
| Theobromine (mg) | 11.05 | 45.91 | 11.05 | 33.15 | 194.69 |

^§^ Sum of (+)-catechin, procyanidin B_2_ and (−)-epicatechin

* Sum of 3-*O*-caffeoylquinic acid, 4-*O*-caffeoylquinic acid and 5-*O*-caffeoylquinic acid

Legend: 1C: group consuming 1 cup of espresso coffee/day; 3C: group consuming 3 cups of espresso coffee/day; PC: group consuming 1 cup of espresso coffee plus 2 cocoa-based products containing coffee twice.

Supplementary Table 2. Analyses of carry-over effect for cross-over treatments. Data reported as the absolute value of carry-over effect size, standard errors were reported in parenthesis. P-values performed according to Bonferroni threshold.

|  | **\|1C - 3C\|** | | **\|1C - PC\|** | | **\|3C - PC\|** | |
| --- | --- | --- | --- | --- | --- | --- |
| **Parameters** | **Effect size** | **^*^P-value** | **Effect size** | ***P-value** | **Effect size** | ***P-value** |
| *Body weight (kg)* | 0.0 (2.8) | 0.999 | 0.0 (2.7) | 0.999 | 0.0 (2.7) | 0.999 |
| *BMI (kg/m^2^)* | 0.0 (0.6) | 0.999 | 0.0 (0.5) | 0.999 | 0.0 (0.5) | 0.999 |
| *Waist circumference (cm)* | 0.2 (1.8) | 0.999 | 0.3 (1.8) | 0.999 | 0.3 (1.7) | 0.999 |
| *Systolic BP (mmHg)* | 1.8 (2.4) | 0.999 | 2.5 (2.2) | 0.999 | 2.5 (2.3) | 0.999 |
| *Diastolic BP (mmHg)* | 0.8 (1.4) | 0.999 | 1.2 (1.6) | 0.999 | 1.2 (1.7) | 0.999 |
| *Total cholesterol (mg/dL)* | 4.1 (6.5) | 0.999 | 2.1 (6.1) | 0.999 | 2.1 (6.2) | 0.999 |
| *LDL cholesterol (mg/dL)* | 0.4 (5.6) | 0.999 | 1.5 (5.4) | 0.999 | 1.5 (5.4) | 0.999 |
| *HDL cholesterol (mg/dL)* | 0.0 (3.4) | 0.999 | 0.6 (3.7) | 0.999 | 0.6 (3.5) | 0.999 |
| *Triglycerides (mg/dL)* | 2.0 (5.6) | 0.999 | 4.4 (5.4) | 0.999 | 4.4 (5.6) | 0.999 |
| *Fasting blood glucose (mg/dL)* | 0.6 (1.1) | 0.999 | 1.2 (1.1) | 0.999 | 1.2 (1.2) | 0.999 |
| *Fasting insulin (mU/mL)* | 2.0 (1.0) | 0.999 | 0.1 (0.7) | 0.999 | 0.1 (0.8) | 0.999 |
| *HOMA-IR* | 0.5 (0.3) | 0.999 | 0.0 (0.2) | 0.999 | 0.0 (0.2) | 0.999 |
| *QUICKI* | 0.0 (0.01) | 0.999 | 0.0 (0.01) | 0.999 | 0.0 (0.01) | 0.999 |
| *NO (μmol/L)* | 0.4 (1.7) | 0.999 | 1.4 (1.7) | 0.999 | 1.4 (1.7) | 0.999 |
| *TMAO (μmol/L)* | 0.3 (0.3) | 0.999 | 0.3 (0.3) | 0.999 | 0.3 (0.3) | 0.999 |
| *IL-8 (pg/mL)* | 0.6 (1.2) | 0.999 | 0.5 (1.3) | 0.999 | 0.5 (1.2) | 0.999 |
| *TNFα (pg/mL)* | 0.0 (0.8) | 0.999 | 0.1 (0.8) | 0.999 | 0.1 (0.8) | 0.999 |
| *VEGF (pg/mL)* | 10.1 (13.7) | 0.999 | 9.1 (14.6) | 0.999 | 9.1 (13.6) | 0.999 |
| **List of abbreviation:** BMI: body mass index; BP: blood pressure; HDL: High-density lipoprotein; HOMA-IR: Homeostatic Model Assessment for Insulin Resistance; IL-8: interleukin-8; LDL: Low-density lipoprotein; NO: nitric oxide; QUICKI: quantitative insulin sensitivity check index; TMAO: Trimethylamine *N*-oxide; TNF-*α:* tumour necrosis factor*-α* ; VEGF: Vascular endothelial growth factor. Legend: 1C: group consuming 1 cup of espresso coffee/day; 3C: group consuming 3 cups of espresso coffee/day; PC: group consuming 1 cup of espresso coffee plus 2 cocoa-based products containing coffee twice per day. *P P-values adjusted using the Bonferroni correction (α’ = 0.05/18 = 0.00278). | | | | | | |

Supplementary Table 3. Analyses of carry-over effect for treatment sequences. Data reported as the time effect and time x treatment interaction coefficients, SEM were reported in parenthesis. P-values performed according to Bonferroni threshold.

|  | **Treatment sequence** | | | | | | **Treatment X time** | |
| --- | --- | --- | --- | --- | --- | --- | --- | --- |
| **Parameters** | 3C/PC/1C | 3C/1C/PC | PC/3C/1C | PC/1C/3C | 1C/PC/3C | 1C/3C/PC | Effect size | *P value |
| *Body weight (kg)* | 0.65 (0.35) | 0.34 (0.21) | 0.39 (0.36) | 0.06 (0.26) | 0.24 (0.25) | 0.25 (0.22) | -0.12 (0.20) | 0.999 |
| *BMI (kg/m^2^)* | 0.20 (0.11) | 0.11 (0.07) | 0.09 (0.13) | -0.01 (0.10) | 0.08 (0.08) | 0.09 (0.07) | -0.04 (0.07) | 0.999 |
| *Waist circumference (cm)* | 0.29 (0.43) | 0.43 (0.41) | 0.04 (0.58) | 0.61 (0.50) | 0.61 (0.38) | 0.18 (0.46) | 0.41 (0.33) | 0.999 |
| *Systolic BP (mmHg)* | -2.50 (2.75) | -3.07 (2.56) | -2.14 (3.63) | 0.57 (3.56) | -0.43 (2.77) | -3.71 (2.44) | 1.68 (2.10) | 0.999 |
| *Diastolic BP (mmHg)* | -0.57 (2.95) | 1.93 (2.34) | -0.64 (2.48) | 0.50 (2.35) | -2.93 (2.69) | -1.57 (2.22) | 2.32 (1.75) | 0.999 |
| *Total cholesterol (mg/dL)* | 3.64 (4.96) | 1.14 (2.46) | 2.43 (4.30) | -0.50 (4.19) | 2.86 (6.19) | 3.29 (4.63) | -2.54 (3.20) | 0.999 |
| *LDL cholesterol (mg/dL)* | 4.64 (4.15) | 3.86 (1.76) | 4.00 (3.04) | 3.36 (2.21) | -1.00 (4.46) | -1.14 (2.97) | 2.18 (2.27) | 0.999 |
| *HDL cholesterol (mg/dL)* | 1.86 (2.46) | -1.21 (1.61) | 0.86 (2.24) | -3.64 (2.40) | 0.00 (2.72) | 1.43 (1.96) | -3.57 (1.59) | 0.476 |
| *Triglycerides (mg/dL)* | -2.29 (4.53) | -6.64 (5.02) | -1.71 (5.01) | -2.00 (6.00) | 0.07 (5.63) | -4.00 (4.90) | -1.46 (3.66) | 0.999 |
| *Fasting blood glucose (mg/dL)* | 1.57 (1.84) | -0.36 (1.66) | -0.29 (1.50) | -3.29 (1.50) | -1.21 (1.69) | -0.14 (1.38) | -1.61 (1.11) | 0.999 |
| *Fasting insulin (mU/mL)* | -0.13 (1.17) | -1.60 (1.11) | 0.09 (1.11) | -3.27 (1.73) | 2.56 (4.83) | 4.45 (4.68) | -4.70 (2.05) | 0.468 |
| *HOMA-IR* | -1.30 (0.76) | -1.92 (0.90) | -0.94 (0.72) | -2.48 (1.24) | 4.12 (4.74) | 5.04 (4.65) | -4.25 (1.98) | 0.576 |
| *QUICKI* | -1.66 (0.73) | -2.01 (1.02) | -1.23 (0.80) | -2.22 (1.23) | 4.56 (4.74) | 5.20 (4.68) | -4.10 (2.00) | 0.630 |
| *NO (μmol/L)* | -0.50 (3.00) | -0.42 (3.23) | -1.47 (2.58) | -2.30 (2.58) | 1.87 (2.24) | 2.78 (2.48) | -2.01 (1.88) | 0.999 |
| *TMAO (μmol/L)* | -0.05 (0.51) | 0.08 (0.51) | 0.21 (0.46) | 0.49 (0.34) | -0.33 (0.57) | -0.47 (0.64) | 0.42 (0.36) | 0.999 |
| *IL-8 (pg/mL)* | -0.56 (1.13) | 1.16 (0.81) | -0.58 (0.91) | -0.28 (0.71) | -1.10 (0.93) | 0.31 (0.76) | 0.57 (0.63) | 0.999 |
| *TNFα (pg/mL)* | -0.15 (0.23) | -0.09 (0.18) | -0.08 (0.23) | 0.03 (0.20) | -1.11 (0.64) | -1.16 (0.62) | 0.59 (0.28) | 0.630 |
| *VEGF (pg/mL)* | -0.32 (7.42) | -9.38 (5.66) | 8.49 (6.87) | 3.87 (5.70) | 1.18 (6.75) | -3.45 (6.81) | -6.14 (4.65) | 0.999 |
| **List of abbreviation:** BMI: body mass index; BP: blood pressure; HDL: High-density lipoprotein; HOMA-IR: Homeostatic Model Assessment for Insulin Resistance; IL-8: interleukin-8; LDL: Low-density lipoprotein; NO: nitric oxide; QUICKI: quantitative insulin sensitivity check index; TMAO: Trimethylamine *N*-oxide; TNF-*α:* tumour necrosis factor*-α* ; VEGF: Vascular endothelial growth factor. Legend: 1C: group consuming 1 cup of espresso coffee/day; 3C: group consuming 3 cups of espresso coffee/day; PC: group consuming 1 cup of espresso coffee plus 2 cocoa-based products containing coffee twice per day. *P P-values adjusted using the Bonferroni correction (α’ = 0.05/18 = 0.00278). | | | | | | | | |

Supplementary Table 4. Post-hoc power calculation for standardized effect size f by treatment comparison. Results reported by Cohen’s f effect size and empirical statistical power estimate (power = 1-β, α = 0.05, n = 21 subjects by group).

| **Parameters** | **Mean (SEM) 1C** | **Mean (SEM) 3C** | **Mean (SEM) PC** | **Cohen’s f** | **Power** |
| --- | --- | --- | --- | --- | --- |
| *Body weight (kg)* | 67.1 (2.8) | 67.1 (2.7) | 67.1 (2.7) | 0.0008 | 0.05 |
| *BMI (kg/m^2^)* | 22.4 (0.6) | 22.4 (0.5) | 22.4 (0.5) | 0.0018 | 0.05 |
| *Waist circumference (cm)* | 73.2 (1.7) | 73.4 (1.8) | 73.7 (1.7) | 0.1242 | 0.13 |
| *Systolic BP (mmHg)* | 113.7 (2.6) | 115.5 (2.1) | 113.0 (2.2) | 0.1404 | 0.15 |
| *Diastolic BP (mmHg)* | 73.1 (1.6) | 72.3 (1.2) | 71.1 (1.9) | 0.0354 | 0.06 |
| *Total cholesterol (mg/dL)* | 166.0 (6.6) | 170.1 (6.3) | 168.0 (5.9) | 0.1398 | 0.15 |
| *LDL cholesterol (mg/dL)* | 87.9 (5.6) | 87.5 (5.6) | 86.0 (5.1) | 0.2686 | 0.44 |
| *HDL cholesterol (mg/dL)* | 65.3 (3.1) | 65.3 (3.6) | 65.9 (3.7) | 0.0889 | 0.09 |
| *Triglycerides (mg/dL)* | 83.1 (6.0) | 85.1 (5.2) | 80.7 (5.5) | 0.0732 | 0.07 |
| *Fasting blood glucose (mg/dL)* | 87.4 (1.2) | 86.8 (1.0) | 85.6 (1.2) | 0.0219 | 0.05 |
| *Fasting insulin (mU/mL)* | 10.8 (1.2) | 8.8 (0.8) | 8.9 (0.6) | 0.0420 | 0.06 |
| *HOMA-IR* | 2.4 (0.3) | 1.9 (0.2) | 1.9 (0.1) | 0.0947 | 0.09 |
| *QUICKI* | 0.3 (0.0) | 0.4 (0.0) | 0.4 (0.0) | 0.1488 | 0.16 |
| *NO (μmol/L)* | 11.7 (1.7) | 11.3 (1.7) | 12.7 (1.6) | 0.1024 | 0.10 |
| *TMAO (μmol/L)* | 2.5 (0.3) | 2.8 (0.3) | 2.5 (0.2) | 0.0098 | 0.05 |
| *IL-8 (pg/mL)* | 9.3 (1.1) | 9.9 (1.2) | 10.4 (1.3) | 0.0533 | 0.06 |
| *TNFα (pg/mL)* | 8.0 (0.7) | 8.0 (0.8) | 8.1 (0.8) | 0.2803 | 0.48 |
| *VEGF (pg/mL)* | 59.1 (11.8) | 69.2 (15.6) | 60.1 (13.5) | 0.1879 | 0.24 |
| **List of abbreviation:** BMI: body mass index; BP: blood pressure; HDL: High-density lipoprotein; HOMA-IR: Homeostatic Model Assessment for Insulin Resistance; IL-8: interleukin-8; LDL: Low-density lipoprotein; NO: nitric oxide; QUICKI: quantitative insulin sensitivity check index; TMAO: Trimethylamine *N*-oxide; TNF-*α:* tumour necrosis factor*-α* ; VEGF: Vascular endothelial growth factor. Legend: 1C: group consuming 1 cup of espresso coffee/day; 3C: group consuming 3 cups of espresso coffee/day; PC: group consuming 1 cup of espresso coffee plus 2 cocoa-based products containing coffee twice per day. | | | | | |
